# Characterizing Pelvic Floor Muscle Activity During Walking and Jogging in Continent Adults: A Cross-Sectional Study

**DOI:** 10.3389/fnhum.2022.912839

**Published:** 2022-06-30

**Authors:** Alison M. M. Williams, Maya Sato-Klemm, Emily G. Deegan, Gevorg Eginyan, Tania Lam

**Affiliations:** ^1^School of Kinesiology, University of British Columbia, Vancouver, BC, Canada; ^2^International Collaboration on Repair Discoveries, Vancouver Coastal Health Research Institute, Vancouver, BC, Canada

**Keywords:** pelvic floor, electromyography, gait, walking, jogging, locomotion

## Abstract

**Introduction:**

The pelvic floor muscles (PFM) are active during motor tasks that increase intra-abdominal pressure, but little is known about how the PFM respond to dynamic activities, such as gait. The purpose of this study was to characterize and compare PFM activity during walking and jogging in continent adults across the entire gait cycle.

**Methods:**

17 able-bodied individuals (8 females) with no history of incontinence participated in this study. We recorded electromyography (EMG) from the abdominal muscles, gluteus maximus (GM), and PFM while participants performed attempted maximum voluntary contractions (aMVC) of all muscles and completed 60–70 strides in four gait conditions: slow walk (1 km/h); regular walk (self-selected comfortable pace); transition walk (self-selected fastest walking pace); jog (same speed as transition walking). We quantified activity throughout the whole gait cycle (%aMVC_GC_) and during periods of bursting (%aMVC_BR_) for each participant, and analyzed the timing of PFM bursting periods to explore when the PFM were most active in the gait cycle. We also conducted a phase metric analysis on the PFM and GM burst timings. We performed a Spearman's rank-order correlation to examine the effect of speed on %aMVC_GC_, %aMVC_BR_, and phase metric score, and used the Wilcoxon Signed-Rank test to evaluate the effect of gait modality, matched for speed (walking vs. jogging), on these variables.

**Results:**

The PFM were active throughout the gait cycle, with bursts typically occurring during single-leg support. The PFM and GM were in phase for 44–69% of the gait cycle, depending on condition. There was a positive correlation between gait speed and both %aMVC_GC_ and %aMVC_BR_ (*p* < 0.001). Phase metric scores were significantly higher during jogging than transition walking (*p* = 0.005), but there was no difference between gait modality on %aMVC_GC_ or %aMVC_BR_ (*p* = 0.059). Where possible we disaggregated data by sex, although were unable to make statistical comparisons due to low sample sizes.

**Conclusion:**

The PFM are active during walking and jogging, with greater activity at faster speeds and with bursts in activity around single-leg support. The PFM and GM co-activate during gait, but are not completely in phase with each other.

## Introduction

The abdominal cavity is the largest hollow space in the body, with the diaphragm rostrally, the pelvic floor muscles caudally, and the abdominal and paraspinal muscles on each side. Contractions of these muscles in isolation or during dynamic movements may increase the pressure (i.e., intra-abdominal pressure, IAP) within the fluid-filled abdominal cavity (McGill and Sharratt, 1990; Neumann and Gill, 2002; Keulenaer et al., 2009). During instances of elevated IAP, the pelvic floor muscles (PFM) contract to prevent downward displacement of the pelvic viscera (Bø and Stien, 1994; Sapsford and Hodges, 2001; Neumann and Gill, 2002; Junginger et al., 2009). Activation of the PFM typically precedes increases in IAP (Pieber et al., 1998; Sapsford and Hodges, 2001), and dysfunctional or absent PFM responses are associated with increased likelihood of urinary incontinence (Pieber et al., 1998; Thompson et al., 2006; Smith et al., 2007). PFM recruitment in response to increased IAP has been demonstrated extensively during discrete and isolated activities such as abdominal contractions (Bø and Stien, 1994; Sapsford and Hodges, 2001; Neumann and Gill, 2002) and during timed breathing or Valsava maneuvers (Bø and Stien, 1994; Neumann and Gill, 2002; Hodges et al., 2007; Park and Han, 2015). However, limited research has explored PFM activity in response to dynamic movements that cause sustained and phasic IAP changes, such as gait.

While resting IAP is in the range of 5–7 mmHg (Keulenaer et al., 2009), walking and running can result in mean IAPs of 12–48 mmHg, with faster speeds causing higher pressures (Grillner et al., 1978; Dietze-Hermosa et al., 2020). IAP is phasic across the gait cycle, peaking during single-leg support in walking, and around heel contact in running (Grillner et al., 1978). At these moments of peak pressure, IAP may reach as high as 73 mmHg (Grillner et al., 1978; Dietze-Hermosa et al., 2020).

Previous work has explored the level of PFM activation during running in continent and incontinent females, focusing on activity around heel contact (Luginbuehl et al., 2013, 2016; Leitner et al., 2017). Data from these studies show that PFM activity increases with increasing speeds (Luginbuehl et al., 2013, 2016; Leitner et al., 2017). The level of PFM recruitment was reported to approach or exceed 100% of the participant's attempted maximum voluntary contraction (MVC) (Luginbuehl et al., 2013, 2016; Leitner et al., 2017), suggesting that the PFM are under considerable strain. As IAP peaks around heel strike during running (Grillner et al., 1978), it is possible that this high activation recorded through electromyography (EMG) corresponds to an increased demand on the PFM to contract against intense IAP changes. However, the extent to which the PFM are active across the entire gait cycle has yet to be reported, and it remains unclear if muscle recruitment is isolated to only the period around heel strike, or if the PFM are active during other phases of gait. Further, activation of the PFM has not been explored at lower, regular walking speeds. While the PFM are likely recruited during walking to counteract smaller increases in IAP (Dietze-Hermosa et al., 2020), it is unknown if the pattern of muscle activation is similar to that of running considering the differing kinematics of gait between these movements. Thus, the purpose of this study was to characterize and compare the timing and amplitude of PFM activity during walking and jogging in continent adults across the entire gait cycle.

## Materials and Methods

The inclusion criteria for participants in this study were healthy, able-bodied males or females between the ages of 19 and 60 years. We excluded individuals if they had abdominal or urogenital surgery within the past 12 months; were currently menstruating or experiencing a vaginal or lower urinary tract infection; or experienced discomfort with walking and standing. We also excluded participants if they experienced urinary incontinence, which we screened for using The International Consultation on Incontinence - Urinary Incontinence Short Form (ICIQ-UI-SF) questionnaire (Avery et al., 2004); we excluded individuals if they scored higher than 0 on the ICIQ-UI-SF.

We based the sample size estimate on previous studies that provided data on changes in PFM EMG amplitude between different intensity conditions during a whole-body activity. When data were reported as medians and inter-quartile range, we converted these values to means and standard deviations as per Wan et al. (2014) and Luo et al. (2018), respectively, in order to calculate effect size. Estimated sample size were based Wilcoxon Signed-Rank test at a desired alpha of 0.05 and power of 80%. Data from Luginbuehl et al. (2016) comparing PFM EMG amplitude during treadmill running at 7 and 11 km/h indicate an effect size of 3.47, yielding an estimated sample size of 3. Data from Saeuberli et al. (2018) comparing average PFM EMG amplitude between drop landings from heights of 15 and 30 cm indicate an effect size of 0.71, yielding an estimated sample size of 15.

We recruited participants using existing lists within our laboratory, as well as through flyers posted in local, public spaces (e.g., coffee shops). All participants attended a single recording session to complete the study protocols in our laboratory at the Blusson Spinal Cord Centre in Vancouver, Canada. The University of British Columbia's Clinical Research Ethics Board approved all procedures, and all participants provided informed written consent.

### Experimental Procedures

#### Electromyography

We affixed surface EMG electrodes (Trigno, Delsys Inc., Boston, USA) to the participants bilaterally over the rectus abdominis (RA; 1 cm lateral and 3 cm superior to the navel), external oblique (EO; 2 cm inferior to the lowest rib on the anterior side), gluteus maximus (GM; half way along the line drawn between the sacral vertebrae and the greater trochanter), and soleus (SOL; 1 cm inferior to the gastrocnemius and 2 cm lateral to midline on posterior shank). To record from the PFM, we affixed a pair of disposable surface electrodes connected to snap-lead Delsys Trigno sensors perianally, ~1 cm lateral to the anus on either side. The research team affixed all sensors to the participants, including the PFM electrodes, which were affixed by a research team member who is a registered nurse. All EMG data were recorded at 2,000 Hz.

#### Attempted Maximum Voluntary Contractions

All participants were novices to PFM training and had no previous, formal instruction in contracting their pelvic floor. A registered nurse with experience in conducting PFM training provided them with a brief education session on the anatomy and function of the pelvic floor, as well as how to contract these muscles. The nurse coached them through multiple pelvic floor contraction attempts using various cues until the participant was able to appropriately contract their pelvic floor without co-activating other muscles. This was confirmed by observing the real-time EMG signals from the PFM and other muscles that may co-activate, as well as by manual palpation of other muscles to which we did not affix EMG sensors (e.g., hip adductors).

Once the participant felt confident in producing a strong pelvic floor contraction and the nurse was satisfied that they could perform the action properly, we recorded EMG from two attempts. All participants performed an attempted MVC (aMVC) of their PFM while lying supine on a plinth with knees flexed and feet flat. Participants maintained the contraction for 4 s. To control for changes in IAP during the contraction, we coached the participants breathing pattern so that an open glottis would be maintained though their contraction: “breathe out (2 s), breathe in (2 s), breathe out (4 s) while contracting your pelvic floor as hard as you can”.

We also recorded two aMVC trials for each of the other muscle groups. For the RA and EO, the participants was positioned in supine with knees flexed and feet flat, and attempted trunk flexion and trunk lateral flexion, respectively. For the GM, the participant lay prone with their knee flexed and attempted to extend their hip so their ipsilateral foot would move toward the ceiling. For the SOL, the participant sat with their leg extended in front of them and attempted plantarflexion. A research team member applied resistance to each maneuver so that all contractions were performed isometrically. We provided participants with as many familiarization attempts as they needed until they reported feeling confident in producing the movement. We also instructed the participants to follow the same breathing technique during each of these aMVCs as described above to control for changes in IAP.

#### Gait Trials

Participants completed 4 gait trials on a treadmill at 3 speeds: (1) *Slow Walk* (1 km/h); (2) *Regular Walk* (self-selected by the participant to represent their usual walking speed); (3) *Transition Walk* (self-selected by the participant as the fastest speed at which they could still walk); and (4) *Jog* (jogging at the self-selected transition walk speed). Participants completed between 60 and 70 strides at each speed. We affixed an infrared-emitting diode to the lateral aspect of the participant's heels and the signal was recorded at 100 Hz using Optotrak Certus position sensors (Optotrak, Northern Digital Inc., Waterloo, Ontario).

### Data Analysis

We analyzed all data using custom-written MATLAB routines (Mathworks, Natick, USA). EMG data were band-stop filtered at 60 Hz, high-pass filtered at 30 Hz, rectified, and low-pass filtered at 500 Hz with a 4th-order dual-pass Butterworth filter. We defined each gait cycle (and the start of the stance phase) by right heel strike, identified by the time at which the heel marker reached its most anterior position. Swing phase was defined using the offset of the soleus EMG signal. We then used these time points to define single-leg and double-leg support phases during gait. We cut the data into individual gait cycles, and then resampled each cycle into 2,000 frames to normalize it in time to 100%. We examined each gait cycle for each participant at each speed and steps with artifact or noise were discarded from further analysis. We discarded all data for a participant for a given condition if they did not have a minimum of 15 acceptable strides. The median number of strides included in the EMG analysis per participant per condition was 55 (range: 18–68); there were only 6 cases where <40 strides were used for analysis. Of the 17 participants, we included 16 (8 F, 8 M), 15 (8 F, 7 M), 13 (6 F, 7 M), and 10 (5 F, 5 M) in the slow walk, regular walk, transition walk, and jog analyses, respectively. The amplitude of each EMG signal was normalized to its respective aMVC in each participant. An ensemble average was created by averaging across all individual participant traces. Ensemble averages by sex were also created for visual inspection.

To determine the timing of PFM activity during the gait cycle, we defined periods of bursting activity for the PFM by any data points that exceeded 2 standard deviations (SD) above the mean of a window of data representing the quietest 5% of the gait cycle. To quantify the amplitude of PFM activity in each participant for each condition, we calculated the mean %aMVC across their entire gait cycle (*%aMVC*_*GC*_) and during their periods of bursting (*%aMVC*_*BR*_).

Because there is some uncertainty about the extent to which PFM EMG signals may be contaminated by crosstalk from the gluteal muscles (Flury et al., 2017), we also quantified the periods of bursting activity in the GM and computed a *phase metric score* to determine the overlap between the timing of PFM and GM activation, following a previous example (Ricamato and Hidler, 2005). We used a threshold of 4 SD threshold above the quietest 5% of the gait cycle to define GM bursts due to more easily definable on and off periods. First, for each participant at each speed, the normalized and averaged EMG profiles of the GMs and PFM were transformed into binary datasets where each frame of the gait cycle was given a score of 1 if the EMG signal was above threshold (muscle active) or 0 if the EMG signal was below threshold (muscle inactive). The binary scores from the right and left GM EMG signals of each participant were then combined and rescored so that 1 represented data points with activity in one or both of the signals, and 0 represented data points with no activity in either signal. To compute the phase metric quantifying the overlap between PFM and bilateral GM activation, we compared the binary scores from the PFM and combined GM signals. The number of frames where the two EMG signals matched [i.e., the two muscles were both active (both 1) or both inactive (both 0)] was expressed as a proportion of the gait cycle. Therefore, a phase metric score of 100% represents a perfect match in the timing of activation between the PFM and GM muscles, and a score of 0% would result if the timing pattern of the muscles do not match at all.

### Statistical Analysis

We used SPSS V.26 (IBM, Armonk, USA) with an alpha of 0.05 to perform all statistical analyses. Descriptive statistics were reported using medians and range. As previous research examining PFM activity during gait has only included female participants, we disaggregated our results by sex when possible. As such, the male, female, and group data is displayed in each figure, as appropriate, and medians/ranges of the outcome measures (%aMVC_GC_, %aMVCBR, and phase metric score) are reported by sex. However, due to the small sample size of individuals included in this study, statistical analyses were only conducted on the combined group data. To determine the effect of gait speed on PFM EMG activity, we plotted the %aMVC_GC_, %aMVC_BR_, and phase metric score for each participant against their treadmill speed for the three walking conditions (slow, regular, and transition walking). We performed a Spearman rank-order correlation on each outcome against speed. [Only the walking conditions (slow, regular, and transition walk) were included in this analysis to control for gait modality]. To examine the effect of gait modality (at matched treadmill speed), we used a Wilcoxon Signed-Rank test to compare %aMVC_GC_, %aMVC_BR_, and phase metric score between the jogging and transition walking conditions. Effect size (*r*) was also calculated for these non-parametric analyses, and *r* of 0.1, 0.3, and 0.5 were considered small, medium, and large effects, respectively (Fritz et al., 2012).

## Results

### Participants

A total of 17 individuals enrolled in this study (8 females, 9 males) with a median age of 27 years (range: 19–44 years). All females were nulliparous, with a median age of 27 years (range: 19–44 years), and a median BMI of 21.3 kg/m^2^ (range: 18.1–30.7kg/m^2^). Males had a median age of 29 years (range: 20–37 years), and a median BMI of 23.2 kg/m^2^ (range: 20.4–30.5 kg/m^2^). The overall median self-selected comfortable walking pace was 2.5 km/h (range: 1.4–3.8 km/h), and the median self-selected transition walking/jogging pace was 5.8 km/h (range: 4.1–7.1 km/h).

### Electromyography

**Figure 1:**
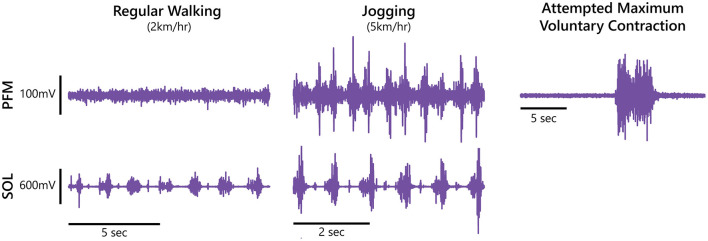
Pelvic floor muscle (PFM) and right soleus (SOL) electromyography recordings during regular walking and jogging, as well as an attempted maximum voluntary contraction of the pelvic floor from a female participant.

Figure 1 presents a sample EMG recording from one female participant during regular walking, and jogging trials, along with the PFM aMVC. The median, raw PFM aMVC value for across all participants was 28.7 μV (range: 7.5–44.9 μV). The median, raw PFM aMVC value for female participants was 29.9 μV (range: 21.0–44.9 μV) and for male participants was 27.3 μV (range: 7.5–33.0 μV).

**Figure 2:**
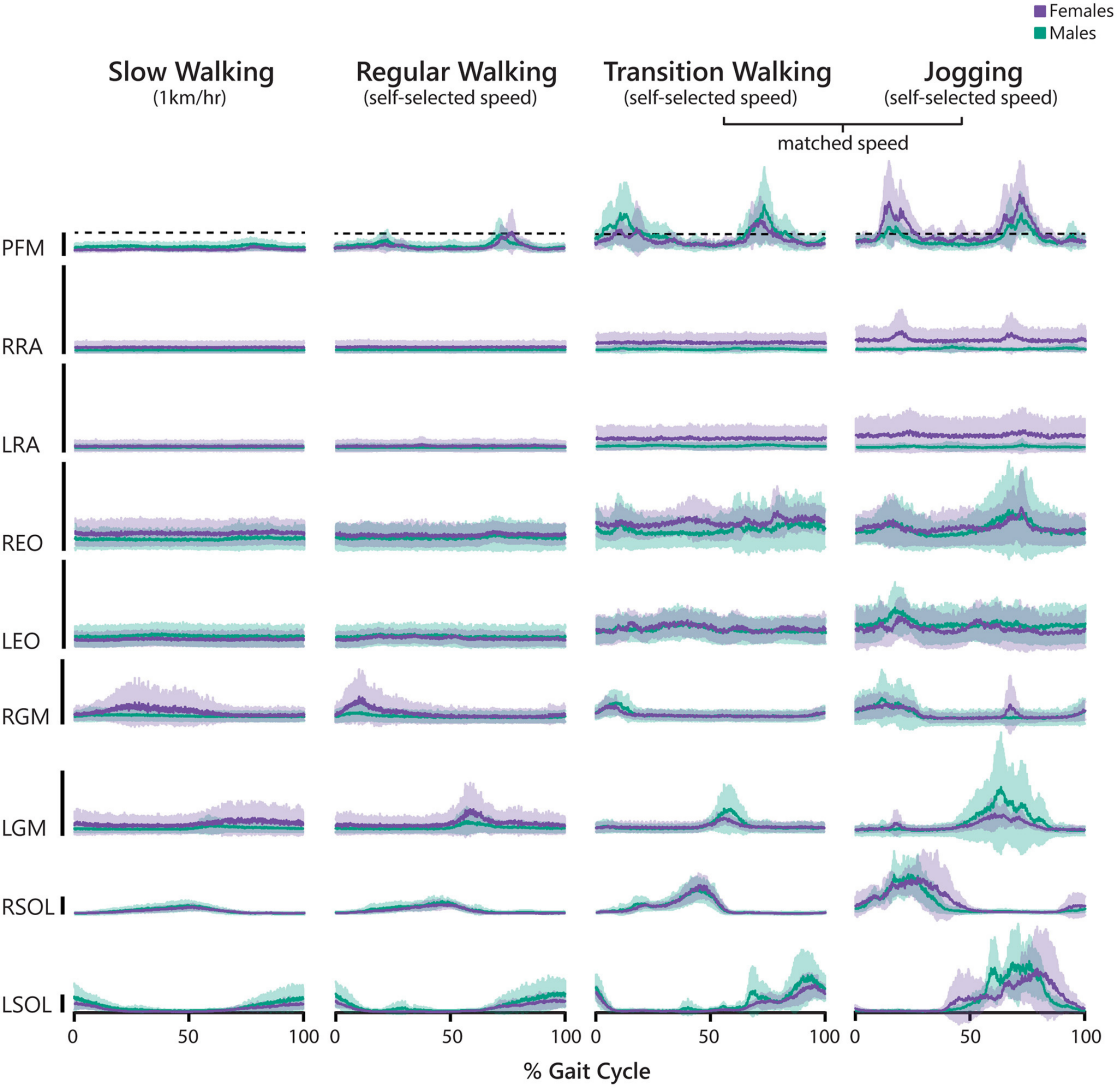
Averaged electromyography (EMG) signals recorded during slow walking, regular walking, transition walking, and jogging normalized to gait cycle (0% indicates right heel strike). Prior to averaging across participants, we normalized each individual trace to the participant's attempted maximum voluntary contraction (aMVC) for that muscle. The black vertical bars represent 100%aMVC for that muscle. Male participants are depicted using green tones, and female participants are depicted using purple tones. Solid lines represent the average EMG activity and the shaded bands represent the 95% confidence interval. PFM, pelvic floor muscles; R/LRA, right/left rectus abdominis; R/LEO, right/left external oblique; R/LGM, right/left gluteus maximus; R/LSOL, right/left soleus.

Ensemble data from all participants (disaggregated by sex) during the four gait conditions are presented in Figure 2. Individual participant data are also available in the Supplemental Material. PFM activity amplitude increased as speed increased, with the emergence of two bursting periods in the fastest 3 conditions. The activation profiles of the other muscles were consistent with what has been reported previously (Lieberman et al., 2006; Anders et al., 2007; Bovi et al., 2011). For female participants, the median %aMVC_GC_ was 23, 35, 72, and 98%, while median %aMVC_BR_ was 25, 51, 81, and 107% at slow, regular, transition and jog conditions, respectively. For male participants, the median %aMVC_GC_ was 31, 40, 82, and 66, while median %aMVC_BR_ was 37, 40, 86, and 81% at slow, regular, transition and jog conditions, respectively. The overall group median %aMVC_GC_ was 26, 36, 77, and 88% during the slow, regular, transition, and jog conditions, and that for %aMVC_BR_ at these speeds was 25, 47, 86, and 99%, respectively. There were no overall differences in %aMVC_GC_ (*Z* = −1.887, *p* = 0.059, *r* = 0.39) nor %aMVC_BR_ (*Z* = −1.886, *p* = 0.059, *r* = 0.39) when comparing transition walking to jogging, but a medium-sized effect was observed for both comparisons.

**Figure 3:**
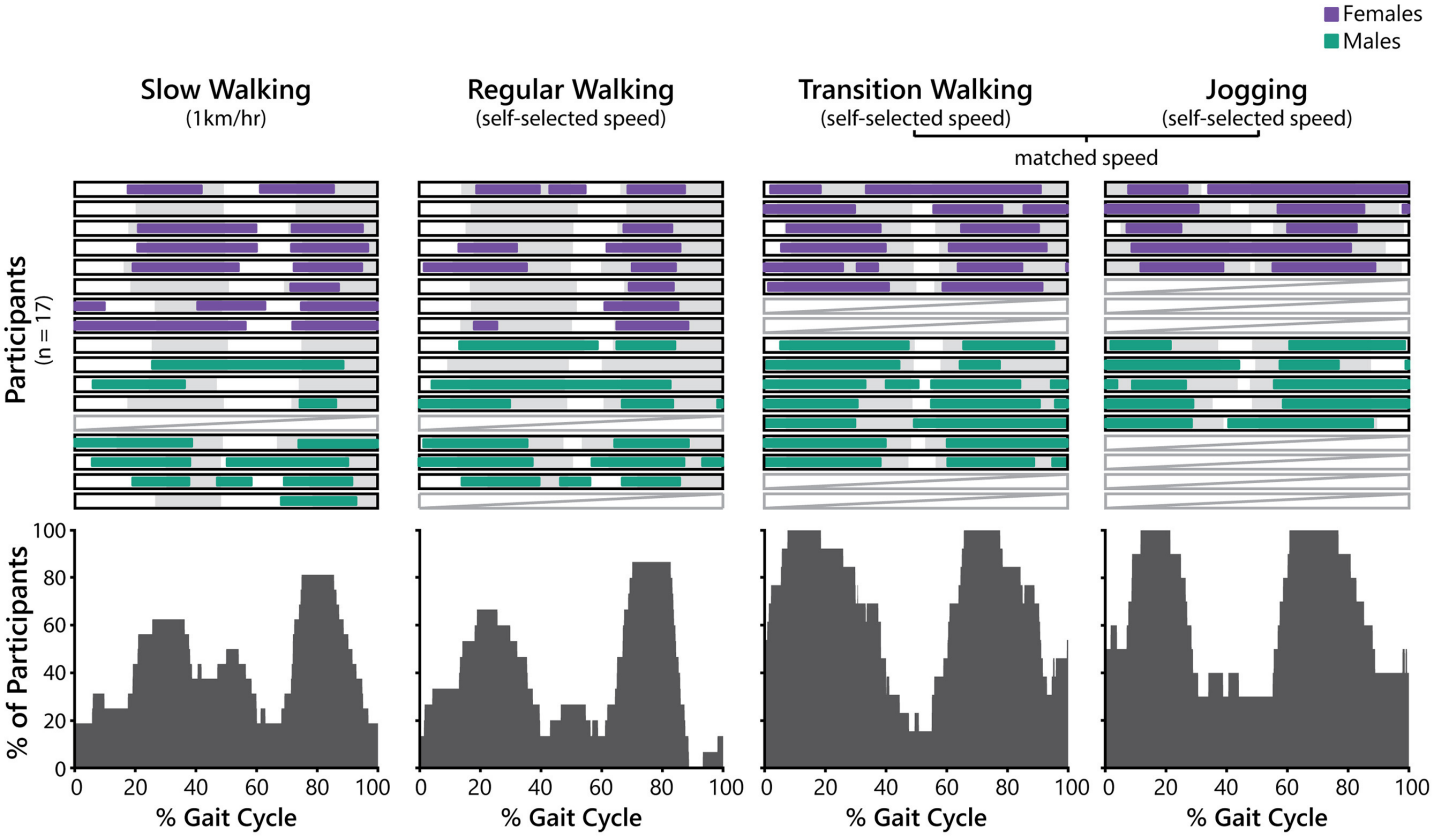
Periods of bursting pelvic floor muscle (PFM) activity during slow walking, regular walking, transition walking, and jogging normalized to gait cycle (0% represents right heel strike). Upper panels: Periods of PFM bursting activity are presented for each participant in each condition. PFM bursting periods are represented by the purple (female participants) and green (male participants) horizontal bars. The gray bars indicate phases in the gait cycle where the participant was in single-leg support. Pale gray boxes with a diagonal line indicates that the data for this participant at this condition was discarded. Lower panels: Summed periods of PFM bursting activity in each condition. Each plot presents the percentage of participants that exhibited PFM bursting for each frame of the gait cycle.

Figure 3 presents the timing of the bursting periods of the PFM during each condition for each participant across the gait cycle. The number of bursts varied between 0 and 3 across participants and across conditions, and there were no apparent differences between male and female participants. In averaging across participants (Figure 3, lower panels), it is clear that PFM activity generally consisted of two bursting periods, which largely corresponded with single-leg support in both walking and jogging.

**Figure 4:**
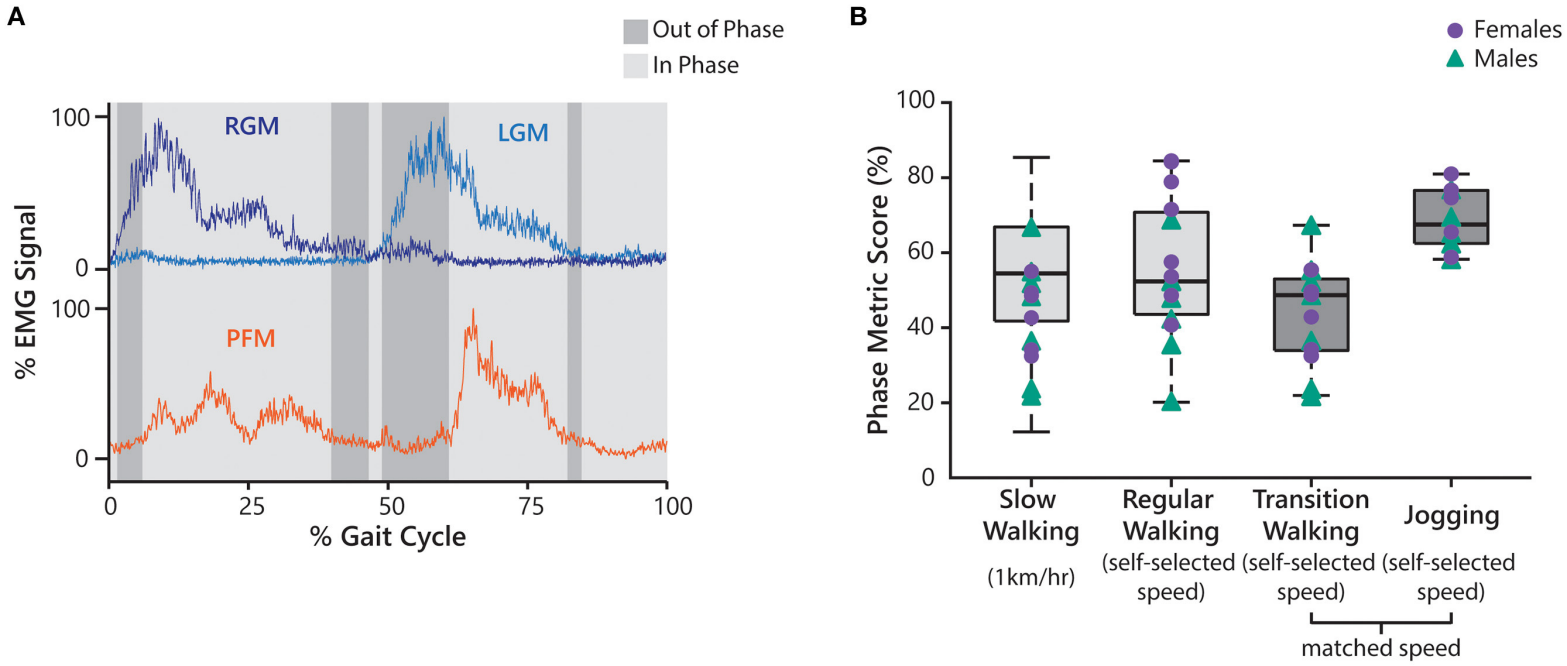
**(A)** An example of averaged pelvic floor muscle (PFM) and right/left gluteus maximus (R/LGM) electromyography activity for one participant during regular walking, normalized to their gait cycle (0% represents right heel strike). Periods of the gait cycle where both muscles are in phase (i.e., both are bursting or neither are bursting) are highlighted in light gray, and periods of the gait cycle where the PFM and L/RGM are out of phase (i.e., only one is bursting) are highlighted in dark gray. **(B)** Results from the phase metric analysis. Individual subject data of female participants is represented by the purple circles, and that of male participants by the green triangles. Only data from transition walking and jogging (dark gray) were used for statistical analysis to compare the effect of gait modality, at a matched speed, on phase metric score.

The phase metric analysis between the bursting periods of the GMs and PFM is presented in Figure 4, with male and female participants represented by different shaped symbols. The median phase metric score for female participants was 53, 64, 46, and 75%, and that for male participants was 60, 48, 49, and 65% for slow, regular, transition, and jogging conditions, respectively. The overall group median phase metric score was 54, 56, 44, and 69% for slow, regular, transition, and jogging conditions, respectively. As such, the PFM and GM were in phase (i.e., both active or both inactive) for approximately half the gait cycle at each speed, but the muscles did not perfectly co-activate. Phase metric scores were significantly higher during jogging than transition walking (*Z* = −2.81, *p* = 0.005, *r* = 0.59).

**Figure 5:**
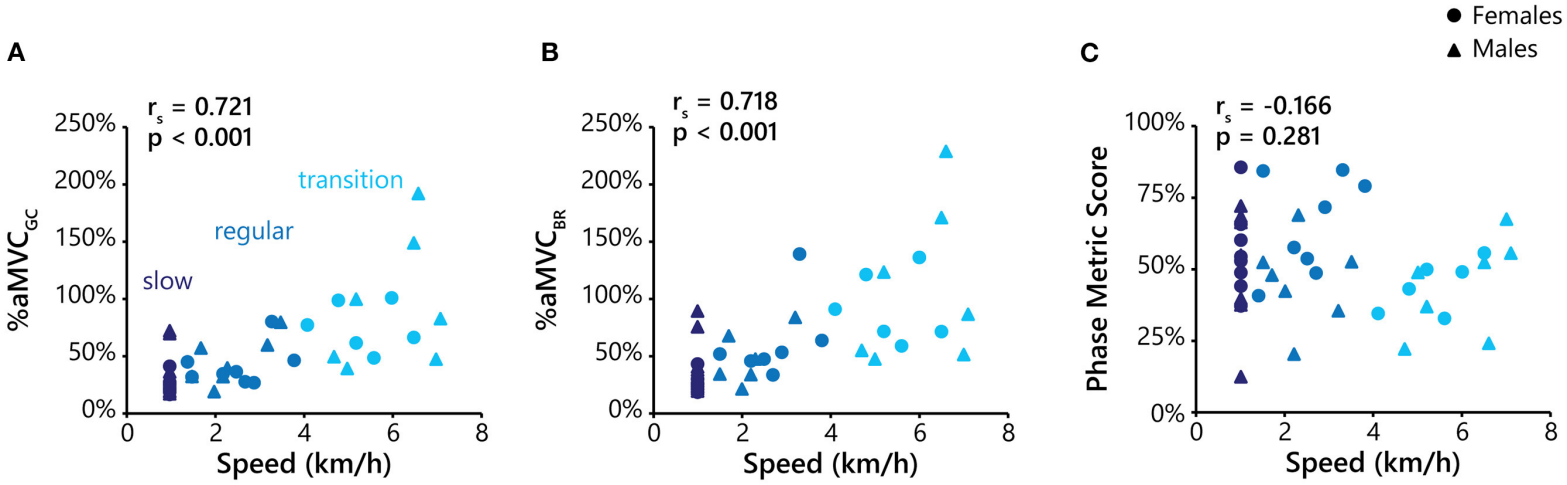
Plots of %aMVC_GC_ **(A)**, and %aMVC_BR_ **(B)**, and phase metric score **(C)** against treadmill speed. For each plot, data is presented from the slow walking (dark blue), regular walking (medium blue), and transition walking (light blue) conditions. Data from female participants are represented as circles, and males as triangles. The alpha value (*p*) and Spearman's correlational coefficient (rho; *r*_*s*_) are displayed in the upper left-hand corner of each plot. Only the walking conditions were included in this analysis to control for gait modality.

The effect of gait speed on %aMVC_GC_, %aMVC_BR_, and phase metric score are presented in Figure 5, with male and female participants represented by different shaped symbols. There was a strong positive correlation between speed with %aMVC_GC_ [*r*_*s*_(42) = 0.721, *p* < 0.001] and %aMVC_BR_ [*r*_*s*_(38) = 0.718, *p* < 0.001]. There was no correlation between speed and phase metric scores [*r*_*s*_(42) = −0.166, *p* = 0.281].

## Discussion

The purpose of this study was to characterize the timing and amplitude of PFM activity across gait speeds and modality. Our results demonstrate that the PFM are active throughout the gait cycle during walking and jogging, with increased activity at faster speeds. Further, PFM activity appears to peak during single-leg support in walking and jogging. The PFM and gluteal muscles co-active to some extent during gait, but are not completely in phase with each other at any speed.

Our results align with and expand previous research that the PFM are active around heel strike during running and that faster speeds increase this activity (Luginbuehl et al., 2013, 2016; Leitner et al., 2017). While we did not measure IAP, previous evidence has demonstrated that IAP peaks during single-leg support in walking and heel contract during running, secondary to increased abdominal activity and vertical loads on the body (Grillner et al., 1978). In our results, PFM bursting generally occurred during the single-leg support phase in both walking and jogging, suggesting that PFM activity may correspond to changes in IAP. However, in addition to these bursting periods where activity often exceeded 100%aMVC, activity of lesser intensity is observed throughout the gait cycle (Figure 2), suggesting that the PFM are consistently active during locomotion. Interestingly, there was no significant difference in %aMVC_GC_ and %aMVC_BR_ between walking and jogging (at matched speeds), despite evidence that jogging should cause greater IAP increases than walking (Grillner et al., 1978; Dietze-Hermosa et al., 2020), which in turn should increase PFM activity. Future research investigating PFM responses during gait should include IAP recordings to explore the intricate relationship between gait speed, IAP, and PFM activity.

Many participants exhibited PFM activity above 100%aMVC during the bursting periods of their gait cycle in the higher speed conditions. PFM EMG activity exceeding 100%MVC was also reported in other studies during running, trampolining, and drop landings (Luginbuehl et al., 2016; Leitner et al., 2017; Saeuberli et al., 2018). It is possible that these high relative values observed in this study are representative of the difficulty that participants had in performing maximum PFM contractions. While participants were given a brief education and training component prior to performing their PFM aMVC, all participants in this study were novices to PFM contractions and learning to voluntarily contract the PFM may be challenging (Bump et al., 1991; Kandadai et al., 2015). Further, previous work has shown that PFM activity is sometimes higher during other exercises, such as trunk flexion or hip extension, compared to the level of activation when an individual is asked to perform an isolated PFM contraction (Neumann and Gill, 2002; Williams et al., 2020). Having participants perform a maximal, isolated PFM contraction may be challenging without extensive training, and therefore may not be a true representation of their maximum. Additionally, we asked participants to perform their aMVC in supine with knees bent and feet flat on a plinth to encourage comfort and a neutral alignment of the pelvis. While there is evidence that bodily position has a significant effect on maximum PFM contraction, there is conflicting information on which posture facilitates the strongest PFM responses. There is evidence for either standing (Chmielewska et al., 2015) or supine postures (Neumann and Gill, 2002; Frawley et al., 2006) producing the strongest PFM contractions, while others claim no difference between supine, sitting, or standing (Bø and Finckenhagen, 2003; Frawley et al., 2005). While normalizing results as a percentage of MVC is effective for making comparisons and giving context to findings, we advise caution when interpreting the level of PFM activation (as a % of MVC) achieved during gait.

There is overwhelming evidence from behavioral and neurophysiological research that the gluteal muscles and PFM share a synergistic relationship (Asavasopon et al., 2014; Yani et al., 2018). Indeed, while people are able to contract their PFM in isolation, it is generally not possible for people to activate their gluteal muscles without co-activating their PFM (Asavasopon et al., 2014; Yani et al., 2018). Considering that the gluteal muscles are active during gait, it should then be expected that the PFM would co-activate during each burst in GM activity. However, the phase metric analysis in this study demonstrated that while PFM and GM bursts do overlap, they are not directly in phase during walking or jogging. Previous research has only explored PFM activation in response to isolated and voluntary gluteal contractions (Bø and Stien, 1994; Asavasopon et al., 2014; Yani et al., 2018), and it remains unknown how this relationship may be altered during dynamic activities like gait where different neural circuitry is involved and the PFM are activated in response to other factors such as modulations in IAP. While our results demonstrated that there was a significant difference in phase metric score between transition walking and jogging, this may be the result of differences in the activation pattern of the GM across gait modalities. During walking, the onset of GM activity occurs after ipsilateral heel strike, but during running, it occurs prior to ipsilateral heel strike (Lieberman et al., 2006). As the PFM appear to be active during similar phases of the gait cycle between transition walking and jogging (Figure 3), it is possible that this shift in GM activity onset may be responsible for the increased co-activation during jogging. The results from our phase metric analysis also support that the PFM signal was likely not contaminated by crosstalk from the nearby gluteal muscles. While the phase metric scores indicate there was a reasonable degree of overlap in bursting between the GM and PFM, there was no condition where these muscles were entirely in phase, meaning that they must represent independent signals.

While we did not conduct statistical analyses to compare the data from male and female participants in this study, visual observation of the disaggregated data (Figures 2–5) indicate no considerable differences by sex with respect to our outcome variables. There is a paucity of research exploring sex differences in PFM EMG signals, with most studies predominantly reporting data from healthy, nulliparous females. One study comparing male and female participants examined the use of a novel EMG anal probe and demonstrated that there was no difference in puborectalis nor pubococcygeus MVC values, but there were differences between males and nulliparous females with respect to anal sphincter MVC (Der Zalm et al., 2013). Our perianal EMG sensors record posterior PFM activity around the puborectalis muscle and external anal sphincter where, anatomically, the PFM may be relatively similar between the sexes. It is possible that EMG activity from the anterior PFM, which support different structures between the sexes, may capture sex-specific differences regarding the recruitment of this muscle group during functional movements.

There are a number of considerations when interpreting these results. First, this study included a small sample of young, healthy adults. Additional work is needed to determine how our findings may translate to older adults or clinical populations. Second, we did not consider habitual physical activity participation among our participants. There is some evidence that physical activity participation could strengthen or weaken PFM function, but it remains inconclusive as to how habitual engagement in high- vs. low-impact activities or sports differentially affects these muscles (Bø and Nygaard, 2020). Future research characterizing PFM activity during dynamic activities may benefit from capturing these data. Noise artifacts in the PFM EMG signals were an issue and data from many strides had to be removed, and entire conditions for certain participants. However, for most participants for each condition, we were able to include the vast majority of strides; in only 6 cases did we accept <40 strides for further analysis. We chose to use perianal surface EMG in this study to improve comfort for participants and facilitate direct comparisons between male and female participants. This decision was informed by our pilot work for this study where the use of vaginal EMG probes was reported to be uncomfortable by female participants to the extent that they felt it could alter their natural gait pattern. The signal from these vaginal probes also appeared to be more susceptible to motion artifacts. By using perianal surface sensors, we could ensure the electrodes maintained good contact with the skin by affixing them with tape, whereas we would be unable to ensure the stability of a vaginal or anal probe position internally. Previous work has also shown no difference in peak or mean root mean squared muscle activity of PFM EMG signals recorded vaginally or perianally (Moretti et al., 2017). However, considering PFM anatomy, the perianal recordings are likely to capture only part of this muscle group and may represent the responses primarily from puborectalis and anal sphincters (Ashton-Miller, 2007).

The results from this study demonstrate that the PFM are active during walking and jogging, with greater activity at faster speeds. The PFM appears to be active throughout the gait cycle, with bursts in activity around single-leg support, when IAP is known to peak. Future research may expand on these findings by recording PFM activity and IAP responses to other forms of gait (e.g., running) and during other dynamic tasks (e.g., jumping) in a wider range of healthy and clinical populations.

## Data Availability Statement

The raw data supporting the conclusions of this article will be made available by the authors, without undue reservation.

## Ethics Statement

The studies involving human participants were reviewed and approved by University of British Columbia's Clinical Research Ethics Board. The patients/participants provided their written informed consent to participate in this study.

## Author Contributions

AW made significant contributions to designing the study protocol, collecting data, analyzing and interpreting data, and prepared the manuscript. MS-K, ED, and GE make significant contributions to data collection, data analysis, and reviewing the manuscript. TL developed the idea for the study, contributed to designing the study protocol, interpreted the data, and revised the manuscript. All authors contributed to the article and approved the submitted version.

## Funding

This work was supported by the Blusson Integrated Cures Partnership and the Canadian Institutes of Health Research (PJT-166040).

## Conflict of Interest

The authors declare that the research was conducted in the absence of any commercial or financial relationships that could be construed as a potential conflict of interest.

## Publisher's Note

All claims expressed in this article are solely those of the authors and do not necessarily represent those of their affiliated organizations, or those of the publisher, the editors and the reviewers. Any product that may be evaluated in this article, or claim that may be made by its manufacturer, is not guaranteed or endorsed by the publisher.
