# Supplementary material for: Characterizing Pelvic Floor Muscle Activity During Walking and Jogging in Continent Adults: A Cross-Sectional Study
Source: Front Hum Neurosci. 2022 Jun 30;16:912839. doi: 10.3389/fnhum.2022.912839 (PMC9279930; doi:10.3389/fnhum.2022.912839)
Supplement: Supplementary file 1 [file Data_Sheet_1.pdf]

	Participant	Sex (M/F)	Treadmill Speed (km/h)	PFM aMVC (μV)	Phase Metric Score (%)	$\%aMVC_{GC}$	$\%aMVC_{BR}$
Slow Walk	2	F	1	36.0	44%	27%	30%
	3	F	1	29.6	37%	25%	26%
	4	M	1	31.8	66%	31%	NaN
	5	M	1	33.0	68%	19%	21%
	6	M	1	28.7	12%	72%	75%
	7	F	1	22.9	54%	22%	25%
	8	F	1	34.1	85%	27%	43%
	9	M	1	10.2	68%	32%	37%
	10	M	1	16.5	37%	36%	40%
	11	M	1	26.0	55%	17%	19%
	12	M	1	27.3	40%	19%	21%
	13	F	1	24.8	49%	41%	NaN
	14	F	1	44.9	60%	16%	18%
	15	M	1	7.5	72%	69%	89%
	16	F	1	30.3	53%	19%	21%
	17	F	1	21.0	66%	20%	23%
Regular Walk	2	F	2.5	36.0	54%	36%	47%
	3	F	3.3	29.6	84%	80%	139%
	4	M	2.2	31.8	20%	32%	33%
	5	M	2.3	33.0	69%	40%	47%
	6	M	3.5	28.7	52%	79%	NaN
	7	F	2.7	22.9	48%	27%	33%
	8	F	1.5	34.1	84%	32%	51%
	9	M	1.5	10.2	52%	32%	34%
	10	M	1.7	16.5	48%	57%	67%
	11	M	2	26.0	42%	19%	21%
	12	M	3.2	27.3	35%	60%	83%
	13	F	1.4	24.8	41%	45%	NaN
	14	F	2.9	44.9	71%	27%	53%
	16	F	2.2	30.3	57%	34%	45%
	17	F	3.8	21.0	79%	46%	63%
Transition Walk	1	M	7	29.2	67%	47%	51%
	4	M	4.7	31.8	22%	49%	54%
	5	M	5.2	33.0	37%	99%	123%
	6	M	7.1	28.7	55%	82%	86%
	7	F	5.2	22.9	50%	61%	71%

	8	F	4.1	34.1	34%	77%	91%
	9	M	6.5	10.2	52%	149%	171%
	11	M	5	26.0	49%	39%	47%
	12	M	6.6	27.3	24%	192%	229%
	13	F	6.5	24.8	55%	66%	71%
	14	F	5.6	44.9	33%	48%	59%
	16	F	4.8	30.3	43%	99%	121%
	17	F	6	21.0	49%	101%	136%
**Jog**	1	M	7	29.2	77%	66%	75%
	4	M	4.7	31.8	65%	60%	71%
	6	M	7.1	28.7	58%	91%	95%
	7	F	5.2	22.9	81%	98%	107%
	9	M	6.5	10.2	62%	110%	133%
	11	M	5	26.0	70%	66%	81%
	13	F	6.5	24.8	77%	86%	94%
	14	F	5.6	44.9	75%	67%	104%
	16	F	4.8	30.3	66%	185%	225%
	17	F	6	21.0	59%	103%	131%

Supplemental Data 1: The phase metric score, $\%aMVC_{GC}$, and $\%aMVC_{BR}$ for each participant during each gait trial that were included in our analyses. Data on sex (M = male, F = female), treadmill speed, and the raw EMG value for each participants' attempted maximum voluntary contraction (PFM aMVC) are also provided.
